# Topics of Public Interest

**Published:** 1918-05

**Authors:** 


### TOPICS OF PUBLIC INTEREST

**The Alvarenga Prize** of the College of Physicians of Philadelphia for 1917 has been awarded to Wilbur C. Davison of Baltimore for his essay on the Superiority of Inoculations with Mixed Triple Vaccine. Essays for the present year must be filed by May 1. The award of about $250, will be made July 14.

**Field Hospitals and Ambulance Companies** will be given odd numbers if supplied with motors, even if the motive power is by horses or mules.

**College Merger.** Bennett and the Chicago Colleges are combined to form the Medical Dept. of Loyola University of Chicago.

**Dependence of Death on Birth Rate.** The paradox that a high birth rate causes a relatively high death rate may be better understood when it is remembered that a quarter of our population is included in about 150 cities of 25,000 and upward. For 1916, they reported about 670,000 live births and 68,500 deaths of infants under one year. Approximately, the 2¾% of the population has a death rate of over 100:1000 population. Thus, when we say that the whole country has a mortality of about 16:1000, it means that the population over one year of age has a mortality of about 13.6:1000. When we realize that for many years, our population has been increased by immigration, mainly by that of healthy, adult males, it is obvious that our total mortality is very much less than it would be, under equal conditions of health and mortality, if our increase in population were dependent solely on births. With the practical cessation of immigration from the middle of 1914 for the period of the war, perhaps for some time after, only a very decided improvement in sanitation and hygiene can prevent a decided rise in our death rate. The general principle involved has a significance in regard to the war that is little appreciated. The various European countries involved, do not depend to any great degree upon immigration for their growth, rather have they lost by emigration. Without being able to furnish exact statistics, it is generally held that the "normal" mortality for a country not markedly affected by immigration and emigration, is nearly 20:1000 though, of course, further improvement is possible. It may even be said that the principle mentioned has a direct military bearing. The U. S. has an advantage in the relatively higher number of adults and, because immigration has generally shown a disproportionate number of males, of man power. Even granting that the excess of male adults is largely at ages beyond those of military service and that it includes alien enemies, there is still a relative excess of man power for industry which probably more than compensates for the weakness of alien enemies of hostile tendencies, and which allows more young men to enter military service without seriously crippling industries and calling women from domestic life, than is the case in Europe. Any excess that the U. S. has in man power, implies a corresponding draft from Europe so that whatever difference exists is double that between a country affected by immigration and one dependent on births. Our relative excess of man power has a further bearing on vital statistics of the future. For various reasons, the birth rate will not be so much diminished in this country. About half the population of any country, is below an age corresponding closely with that of maturity. This part of the population is, on the whole, more vulnerable, especially to the indirect influence of war and it becomes more and more vulnerable as we descend in age. The European countries not only have a relative excess of the vulnerable part of the population—say 52 or 53¾ as against 48 or 47% for the U. S.—but for all except France, i.e. for all which have hitherto shown a healthy growth of population, there is a disproportionate excess of the lower and lower age groups of increasing vulnerability. It has been said, for example, that in Poland. at least in those parts pretty directly subjected to military operations, the population under five years of age has been practically wiped out. The same principle is illustrated, though to a less degree, in Belgium. With these exceptions, it is undoubtedly operative to a greater degree in the central powers, than in their antagonists. Under conceivable estimates of the duration and local incidence of the war, it is apparent that, relatively speaking at least, the U. S. will have an enormous advantage over all the other belligerents, a most favorable omen for the future.

---

**The Franking Privilege** is accorded to all sailors, soldiers, doctors and nurses at the front. It is even proposed to extend it to all in the military service, even in this country.

---

**Attrition of the War.** The total British losses on the western front were 129,089 for Nov., 79,527 for Dec., 73,017 for Jan. The proportion of killed to wounded varies from 1: 3 and a fraction up to 1: 6, by weekly reports but the latter ratio occurs when the missing are an important element, as in more active hostilities with change of position. About 25% of wounds incapacitate for further military service, at least for a considerable time. Thus, aside from prisoners, the definitive losses are just about double the direct fatalities and range from 20 to 40% of the total casualties, averaging about 33.3%. If the quarter year is taken as a fair standard, the definitive losses of the British are at the rate of about 375,-000 a year. This is for about 135 miles of the total 460 miles of the western front, the Belgians occupying 16 miles and the French the remainder until recently, the proportionate definitive losses for the entire front being about 1,200,000. It is probable that the German losses are about equal. The recent French estimates of the German forces are: Western front, 150 divisions, 2,250,000 men; eastern front, 79 divisions, 1,185,000 (many of which are believed to be moving toward the west); Italian front, 7 divisions and 4 in reserve, 165,000; total 3,600,000. This agrees quite well with the estimate published some months ago, that the total initial military strength of Germany was about 7,000,000, of which the definitive losses for the first years were slightly over 3,000,000. Indeed, allowing for the half year of hostilities since Aug. 1, 1917, the agreement is surprisingly close. The large excess of man power among the Entente Allies and the full occupation of Germany's allies with their own problems, indicates that, even if Germany need to hold only the western front and have no serious economic problems, attrition alone will bring the war to an end in about 3 years more. Of course, the western front can be diminished in length but more by straightening salients than by retreat to any logical line which does not in itself virtually concede the issue of the war. Any advantage gained in this way will be equally advantageous to the Entente Allies and will scarcely diminish the casualties though it will, of course, lengthen the period required to complete the war by attrition. Actually, we shall be surprised if the war continues to the time limit of attrition. Such a contingency would be too highly analogous to the battle between the ram and the maul which continued till the handle of the latter and the tail of the former were worn down to a sliver and a hair respectively.

One set of statistics, estimates Germany's militia at 14 million—20% of the total population—the mobilization at 10,650,000 including the class of 1919, the definitive losses at 4 million or at 6 million according to French statements and also those by a socialist member of the Reichstag. Now it is undoubtedly a fact that Germany did have 14 million men registered in military units and well trained but the general military axiom remains true that no country can maintain in the field more than 10% of its total population. The utilization of female labor and especially the labor of occupied countries undoubtedly gives Germany an advantage over most countries in this respect but a study of population by sex and age groups shows that, for various reasons, even these factors cannot increase the actual fighting forces much above the 10% standard except for a very brief period. To a large degree, the apparent contrast between Germany and her opponents lies merely in the fact that transportation and manufacture of a military nature as well as heavy toil generally is done by men technically listed as soldiers in the one case and as civilians in the other. The higher figures of definitive losses probably represent the inclusion of deaths and disablement not directly due to military casualties. It is simpler to ignore these and to accept the general principle that the recruiting of an army by the annual classes of youths will just about equalize non-military losses, just as the increase of the population as a whole cannot be increased by the uprising generation, to any appreciable degree, within any period of a few years which may be regarded as the maximum duration of any modern war of great magnitude. To mobilize now, the class of 1919, is not only in a general sense drawing on interest not yet accrued but, while it may of course tide over a short period, it means a disproportionate susceptibility to both military and non-military losses.

It is interesting to note that Austria-Hungary, with a population of about 50,000,000 and an initial potential militia of about 5,000,000, is now estimated to have a force of only 1,239,000, whereas, proportionately to the first estimate of Germany's remaining man power, she should have about 2,570,000. The discrepancy may be exaggerated but it is true that the losses from all causes have been greater than for Germany, especially in prisoners, due perhaps to the lack of cohesion among the component national groups of the dual empire. Then, too, Austria-Hungary has lost considerably by disease of the older camp types, due to lesser sanitary efficiency.

---

**Anthrax** was discovered Feb. 1 in a cow near Attica Center.

---

**The Shrinking Dollar.** A certain type of statistics shows that the dollar, averaging 100 cents worth of purchasing power 1900-1906, as against 126 cents from 1896-1898 declined to 82 in 1914, 66 in 1915, 51 in 1916 and is now down to about 45 cents. This is undoubtedly correct for certain commodities, foods in particular but, after all, foods do not comprise a very large part of the budget for the average family and the very poor laborer is getting about 250 cents for every 150 he got in 1896-8.

---

**Newspapers** are suggested as a backing for absorbent pads, instead of gauze, as a war economy.

---

**Smoking by Nurses** is discussed by a correspondent of the Trained Nurse, Feb. 1918. Smoking by women in England and various continental countries—though not France—is considered quite proper though army nurses are supposed not to smoke while in uniform nor while on duty. It is an open secret that many respectable American women smoke, though usually surreptitiously. It is regarded as quite probable that the use of tobacco by women will be increased during the war and some interesting social problems are suggested.

---

**Simple Method of Estimating the Depth of a Foreign Body.** Capt. A. Howard Pirie, a Canadian physician, compares the measurements of the shadow on plates at the surface of the body and at a known distance the calculation of the depth being easily made on the principle of proportionate triangles.

---

**Volunteer Medical Service Corps.** The Council of National Defense intends organizing for home service, the entire medical profession not available for active service. Due notice will be received by physicians and we trust that this locality will respond promptly and unanimously.

---

**Protracted Gestation in Relation to Military Service.** A captain in the R. A. M. C. sued his wife for divorce on the ground that she gave birth to a child 307 days after his departure. Testimony elicited histories of gestation of 298, 306 and 308 days under similar circumstances. Contradictory opinions were expressed and, as usual, the wife was given the benefit of the doubt.

---

**Court Martial for Neglect of Meningitis Case.** In regard to the press notices of a case and others of similar nature, we ask our readers to reserve decision till the findings of the court are published. The preliminary official report shows that the complaint that the body of the soldier was shipped wrapped in a sheet was not well taken as this is the proper procedure in infectious cases. The "deplorable" condition of the hospital is also admittedly due in part to lack of completion for which the medical officers were not responsible.

The court martial can be relied upon to do full justice to all concerned.

---

**Veterinary Mal-Practice Suit.** Damages were awarded in court at Lockport, Mch. 6, to a farmer of Niagara Co. to the amount of $125 against a Buffalo veterinarian, for the loss of a colt.

---

**Centenarians.** Mrs. Sara Green died Mch. 6 in Ceres Township, near Bradford, Pa., aged 102 less 14 days. Mrs. Sally Gold of Williamsburg cast her first vote in March, at the age of 102. Incidentally, we may express a prejudice against any locality that holds an election on any date except the first Tuesday after the first Monday in November.

---

**Cures by Quacks.** Newspapers quite generally publish testimonies of this sort, with full names, street address and portrait. The testimony is direct, positive, unequivocal. It is the experience of the "masses" addressed to the masses. It can be assailed only on the basis of its ultimate truth. Is it not worth while for individual physicians to pay attention to this evidence and to meet it with equally direct, positive and unequivocal statements? Will our readers help?

---

**Conserve Glycerin, Sugar and Alcohol.** The U. S. Food Administration asks physicians to avoid the use of these substances in prescriptions so far as possible, even by selecting Galenicals that do not contain them.

---

**Municipal Health Center.** The fifth Buffalo institution of this kind has been opened in the old Tracy house at the corner of Court and Franklin Sts. Dr. Chas. Leone, city physician, will hold office hours at 8:30, 1-3 and 7-8. All persons needing free medical treatment—and we trust that a rigid exclusion of undeserving cases will be made—may apply in person or by telephone. Miss Laura M. Hamilton is the nurse in charge. Venereal clinics, especially for soldiers, will be held from 3 to 4 and 7 to 8 daily. Clinics in infant welfare, tuberculosis, for the feeble minded and dental, will be held 2-3 times a week.

**Quartermaster Supplies for Officers.** A bill has been introduced into congress, providing for the issue of uniforms and other equipment to officers. The object of the bill is not only to protect officers from profiteering but to secure uniforms that are uniform and to prevent the marked tendency to get as far as possible from standard tints, designs and measurements as possible without having the equipment condemned.

---

**Statistics of Medical Dept. of the Army.** March 15. (Regular Medical Corps, 799—1 major general, 66 colonels, 102 lt. colonels, 177 majors, 2 captains, 451 lieutenants. Medical Reserve Corps, 18,037—1,085 majors, 4,163 captains, 13,789 lieutenants; on active duty, 14,655—937 majors, 3,579 captains, 10,139 lieutenants. Medical Corps, National Guard, 1,242—11 lt. colonels, 257 majors, 151 captains, 825 lieutenants. Medical Corps, National Army, 76—4 brigadier generals, 12 colonels, 55 lt. colonels, 5 majors. (This last is not in anyway limited to the supply of medical officers to the National Army, indeed medical officers are distributed according to convenience. The National Army medical corps seems to be used mainly for promotion of deserving officers when there is some obstacle to their promotion in one of the other corps and when the limitation to the period of the war is not a personal objection—and why any doctor would want to stay in the military service after the war is a mystery to most reserve men). Dental Corps, 210. Dental Reserve Corps, 5,124 of whom 1,337 are on active duty. Veterinary Corps 24. Veterinary Reserve Corps, 1,421, of whom 794 are on active duty. Veterinary Corps, N. G. 57. Veterinary Corps N. A. 297. Sanitary Corps 947. Ambulance Service 142.

---

**The Alumni of the Medical Dept., University of Buffalo,** will hold their 43d Annual Meeting June 5-7. Reunions of classes whose dates end in 3 and 8 will be held on the first day. Details will be issued later. Dr. Wm. F. Jacobs is Chairman of the Executive Committee.

---

**Censorship of Articles by Members of the Medical Reserve Corps.** By order of the Surgeon General, all medical officers publishing papers, will submit copy in duplicate to his office, for permission, upon receipt of which one copy wll be forwarded to the journal designated. This is not a new regulation but is in accordance with paragraph 423 of the Manual of the Medical Dept., and applies to professional papers requiring reference to official records or based on experience gained in the discharge of duties. This journal has already complied with the spirit of this regulation and will enforce the letter.

---

**Centenarians.** Mrs. Kitty Brown Sutton, the oldest inhabitant of Orleans Co., N. Y., died at Two Bridges, Apr. 2, aged

100 less 1 month and 21 days. She was a native of London, Eng. Mrs. Hannah G. Leslie, the oldest inhabitant of Chautauqua Co., died at Jamestown, N. Y., Apr. 8, aged 100 years, 3 months, 8 days.

---

**Births and Deaths of Buffalo.** March 1918, respectively 1303 and 688. March 1917, 1272 and 772. First quarter of 1918, 3367 and 1954.

---

**Casualties of American Army in France.** For entire period up to April 2, 1918, 183 killed in action, 52 of wounds, 39 of gas or unknown causes; accidental deaths 164, by disease 793, by loss at sea, mainly from Tuscania, 793. Total deaths 1476. There were also 829 non-fatal wounds, 22 captures and 41 missing.

---

**Summer School.** Dr Chas. Bernstein, Supt. of the Rome Custodial Asylum, announces a summer course for teachers of the feeble minded in public day schools and institutions, July 1-27. Both didactic and practical instruction will be given. The only charge will be $20 for board and lodging.

---

**The National Board of Medical Examiners** is holding a series of examinations for physicians at the training camps, a typic session including 5 days of written examinations and 6 of practical work in laboratories and clinics. The examination is said to confer the right to practice in all states and to take the place of formal examination for the government services ,and it is expected that international reciprocity will be secured, at least for the Entente Allies.

---

"**Milk for Babes.**" The Children's Bureau of the Dept. of Agriculture, from a study of 756 children aged 2-7 in Baltimore, finds that only 29% are now having fresh milk, as against 60% last year, and that only 3% are having as much as three cupfuls a day. As milk production is very directly influenced by the higher price of labor and the demand for cereal products, live stock, etc., it is now declared by many authorities that the minimum retail price of high-grade milk cannot be much less than 12c a quart, as against 5 or 6c for the ordinary commercial—and rather dangerous—product of a generation or less ago. While public schemes of subsidizing dairy industries or of furnishing milk to the poor below cost naturally suggest themselves, it is worth while considering whether it should not be put plainly before the public as a patriotic duty to do away with the use of milk as a beverage for healthy adults. Milk is necessary in the preparation of various foods and for the nourishment of children and the sick; it is not a well balanced food for adults, nor economic for this class. Yet an enormous quantity is consumed in addition to an abundance of other foods.

---

**Wheat Conservation by the Army.** The white wheat bread of the U. S. Army is about the best product of the kind known, whether from the standpoint of nutrition or routine palatability. But the various combination flours and corn preparations, especially the nearly white corn meal of the south, are only slightly less valuable as food and are very palatable as a change. Soldiers generally relish the change, and there seems to be no reason why one or two wheatless days or several wheatless meals a week should not be adopted for the cantonments of this country at least.

---

**Arsphenamine and Neoarsphenamine.** The Hygienic Laboratory of the Public Health Service, Washington, noting the reports of unfavorable results from the use of American-made salvarsans, desires to test all samples that have appeared clinically to be unsatisfactory. Care should be taken to send ampoules of the same lot number as the one used. The patient's weight and age, dose and dilution employed, and clinical data of symptoms and results should accompany the sample.

---

**Food Conservation as Indicated by Garbage.** The diminution of garbage for N. Y. City for the first half of 1917 was only 9.16% (as compared with the last half of 1916) which is not unusual. 62 of 81 smaller cities showed reduction of the September garbage, this being the month when canning is usually at its height. However, it may be questioned whether this represents a genuine economy or a diminution of canning owing to high prices and the difficulty of securing sugar. Several cities have showed a notable diminution in garbage and nearly all waste of fats. Washington yielded 104.8% and Los Angeles 101.1% of the normal amount of garbage, but both have increased in population, the former markedly. The general impression is that while a considerable amount of food is still utilized that was formerly wasted, much further saving is possible. In this connection it may be mentioned that there is a strong tendency in military cantonments to reduce incinerators to a minimum, as wasteful of food, garbage that can be fed to pigs, metal and paper that can be re-used, as well as of fuel and of labor for their maintenance and for cleaning soot. In many instances, however, there is no local market for camp waste, even for manure to be hauled away free. Thus the attempt to conserve waste costs more than destruction.

---

**More Medical Reserve Officers Needed.** April 8 there were on duty 15,174 Medical Reserve Officers (beside a few thousand not called additions to the regular Medical Corps, and National Guard and National Army surgeons). There is at present a comparatively small excess of surgeons immediately available and the Reserve Corps on duty is just about equal to the needs of the 1,500,000 force that will undoubtedly be in Europe before the close of the year. At a conservative estimate 15,000 more members of the Medical Reserve will be needed in the near future. Let us emphasize the fact not realized by many physicians, nor by the public generally, that the country needs, not a spontaneous patriotic uprising of surgeons or soldiers, without training, at the moment when an emergency has already arisen, but a farther-sighted patriotism that will provide an ample supply in advance of an emergency, and that will allow careful selection and training (three months at least). The complacent view of meeting an emergency when it occurs is due largely to reiterated false teaching about the Revolution. No subsequent war of the U. S. represented such adequate and prolonged preparedness. There had been universal militia training for the entire colonial period, with actual experience in war during the whole of the 18th century, and there was a large body of French and Indian War veterans, still in the prime of life available. Furthermore, so far as was possible without a formal government, and indeed, contrary to the existing governments, there had been preparation of an active nature for at least ten years.

